# A high-throughput single-particle imaging platform for antibody characterization and a novel competition assay for therapeutic antibodies

**DOI:** 10.1038/s41598-022-27281-w

**Published:** 2023-01-06

**Authors:** Elif Seymour, M. Selim Ünlü, John H. Connor

**Affiliations:** 1 Department of Biomedical Engineering, Boston University, Boston, MA 02215 USA; 2 Department of Electrical and Computer Engineering, Boston University, Boston, MA 02215 USA; 3 Department of Microbiology, Boston University School of Medicine, Boston, MA 02118 USA; 4 Present Address: Lunenfeld-Tanenbaum Research Institute, Sinai Health System, Toronto, ON M5G 1X5 Canada

**Keywords:** Microbiology, Biomedical engineering, Assay systems, Infectious diseases

## Abstract

Monoclonal antibodies (mAbs) play an important role in diagnostics and therapy of infectious diseases. Here we utilize a single-particle interferometric reflectance imaging sensor (SP-IRIS) for screening 30 mAbs against Ebola, Sudan, and Lassa viruses (EBOV, SUDV, and LASV) to find out the ideal capture antibodies for whole virus detection using recombinant vesicular stomatitis virus (rVSV) models expressing surface glycoproteins (GPs) of EBOV, SUDV, and LASV. We also make use of the binding properties on SP-IRIS to develop a model for mapping the antibody epitopes on the GP structure. mAbs that bind to mucin-like domain or glycan cap of the EBOV surface GP show the highest signal on SP-IRIS, followed by mAbs that target the GP1-GP2 interface at the base domain. These antibodies were shown to be highly efficacious against EBOV infection in non-human primates in previous studies. For LASV detection, 8.9F antibody showed the best performance on SP-IRIS. This antibody binds to a unique region on the surface GP compared to other 15 mAbs tested. In addition, we demonstrate a novel antibody competition assay using SP-IRIS and rVSV-EBOV models to reveal the competition between mAbs in three successful therapeutic mAb cocktails against EBOV infection. We provide an explanation as to why ZMapp cocktail has higher efficacy compared to the other two cocktails by showing that three mAbs in this cocktail (13C6, 2G4, 4G7) do not compete with each other for binding to EBOV GP. In fact, the binding of 13C6 enhances the binding of 2G4 and 4G7 antibodies. Our results establish SP-IRIS as a versatile tool that can provide high-throughput screening of mAbs, multiplexed and sensitive detection of viruses, and evaluation of therapeutic antibody cocktails.

## Introduction

Diagnosis and therapy of viral infections are important public health concerns as it is revealed dramatically by the latest COVID-19 pandemic. The novel coronavirus that caused this pandemic, SARS-CoV-2, was originated in Wuhan, China in December 2019 and has since spread around the world, leading to a major global crisis with over 6 million deaths as of July 2022^1^, and damaging economies and social life. Moreover, recent monkeypox outbreak that occurred in multiple countries, including U.S. and Canada, further established the importance of readiness to respond to outbreaks^2^. One component of an effective response to an outbreak is fast and accurate diagnosis of the viral infection. Current laboratory tests for viral diagnostics are mainly based on polymerase chain reaction (PCR). Although PCR-based tests are reliable and sensitive, due to their multi-step workflows, they require special equipment, trained personnel, and laboratory environment. On the other hand, rapid diagnostic tests have the advantage of being performed by minimum sample processing in a short period of time at the point-of-care (POC). Biosensors have been shown to provide rapid and sensitive platforms for virus detection using a variety of transduction mechanisms and became ideal candidates for rapid viral diagnostics due to their ease of use, portability and miniaturization through microfluidic and microarray technologies^3,4^. One such biosensor platform, developed by our group and termed as Single-particle Interferometric Reflectance Imaging Sensor (SP-IRIS), can detect individual viral particles captured on an antibody microarray printed on silicon/silicon dioxide (Si/SiO_2_) substrates without use of labels^5,6^

Compared to traditional dark-field imaging where the signal is obtained only from the scattered light from the object of interest, the biggest advantage of SP-IRIS lies in the interferometric component of the signal which results from the interference between the scattered light and the light reflected from the Si/SiO_2_ interface. As the object size decreases to nanometer range, the scattered light intensity decreases greatly whereas the interference signal can be orders of magnitude larger than the scattered intensity, enhancing the contrast of nanoparticles^7^. One other major advantage of SP-IRIS is that only surface-bound particles are imaged on the camera; therefore, the signal does not vary depending on the media that the sample is in, allowing use of complex media and different conditions^8^. Signal dependence on media is an inherent problem of surface plasmon resonance (SPR) based sensors. The background signal resulting from the changes in the refractive index of the solution, known as bulk effect, affects the sensitivity and accuracy of measurements^9^. SP-IRIS also has a very robust mechanism of detection that is independent of temperature fluctuations or binding position of the nanoparticles on the sensor surface as opposed to the optical resonator-based sensors where the signal-to-noise ratio changes based on these parameters^10^. In addition, for both photonic crystal and nano-resonator based sensors, active sensor area is very small, therefore, at low concentrations, binding events are very rare, limiting the sensitivities of these systems^11^. Thus, SP-IRIS is distinguished from other label-free optical techniques with its high sensitivity, robustness, and simplicity^12,13^ SP-IRIS has other capabilities such as providing size and shape information as well as individual nanoparticle tracking which is valuable for differentiating between low and high affinity particles^14^. Moreover, recent microfluidic integration advanced SP-IRIS to a sensitive, real-time immunoassay platform through disposable cartridges, suggesting its great potential for POC diagnostics applications^15–17^.

A key component of multiplexed immunoassay design is the selection and characterization of the capture antibodies. Monoclonal antibodies (mAbs) are the most commonly used capture probe type in immunobiosensors owing to their specificity and homogeneity. Selecting the proper capture antibodies requires a careful evaluation of the binding parameters such as affinity and specificity. An ideal capture antibody should have a high affinity and specificity for the target and show minimal cross-reactivity against other species. Use of microarray-based substrates and high-throughput readout capability make SP-IRIS a suitable platform for screening a large number of capture antibodies on a single chip. Here, we utilize SP-IRIS platform for the selection of high-affinity capture antibodies against two species of ebolavirus (Ebola and Sudan viruses, EBOV and SUDV), and Lassa virus (LASV) using recombinant vesicular stomatitis virus (rVSV) models. We present the performance of 30 mAbs on SP-IRIS for the detection of whole rVSV particles expressing EBOV, SUDV and LASV glycoproteins (GPs). We also propose a model for GP epitope mapping based on the level of the binding that occurs on SP-IRIS.

In addition to diagnostics, another critical aspect of a fight against a pandemic is the development of effective therapeutic agents. Monoclonal antibody-based therapies offer a promising approach for early intervention against viral infections due to their safety and specificity^18^ Moreover, with the recent advancements in mAb screening and identification, highly potent mAbs can be developed rapidly and progress into the clinical trial phase within the order of months^19^ Multiple mAbs against Ebola virus GP have been identified so far^20–23^ The antibodies that showed protection in rodent and/or guinea pig models have been selected for further studies involving EBOV-infected non-human primates (NHPs). Among these, KZ52, which is a neutralizing antibody isolated from a patient who recovered from EBOV infection in the 1995 Ebola outbreak (Kikwit, Democratic Republic of Congo), showed full protection in guinea pig models, however failed to provide protection in NHP models when administered as a single antibody treatment^24^ In a different study that used a combination of two mAbs, ch133 and ch226, partial protection was achieved in NHPs and it was suggested that combining three or more mAbs might improve the protective efficacy especially if they target different epitopes on the glycoprotein hence combining different mechanisms of action^25^. This hypothesis was supported further by another study where a cocktail of three mAbs, selected from eight mAbs based on the results of single antibody treatments, was found to be fully protective when it was given to guinea pigs 2 days after infection while these antibodies were less protective when administered individually^26^. This antibody mixture, called ZMAb, is composed of 1H3, 2G4 and 4G7 mAbs, and was also demonstrated to have 100 and 50% efficacy when the treatment was given to cynomolgus macaques 1d and 2d after exposure, respectively^27^. Another mAb cocktail that was tested in NHPs was named MB-003 and consisted of 13C6, 13F6, and 6D8 antibodies. This antibody mixture provided significant protection in rhesus macaques when administered 24 or 48 h post-infection^28^, and it was also shown to be successful when the treatment started after the onset of the symtpoms^29^. Some of the components from ZMAb and MB-003 cocktails have been combined to create a third antibody cocktail, titled ZMapp, that was shown to have an improved efficacy compared to the former two cocktails^30^. ZMapp was also shown to be beneficial in a randomized, controlled study in patients with Ebola virus disease during the 2014 Ebola epidemic in West Africa^31^.

ZMapp contains 2G4 and 4G7 from the ZMAb mixture and 13C6 from the MB-003 cocktail. A study by Murin et al. provided single-particle electron microscopy reconstructions for the mAbs in the ZMapp cocktail as well as the additional 1H3 mAb^32^. They showed that 13C6 and 1H3 bind overlapping epitopes in the glycan cap and compete. They also showed that 2G4 and 4G7 bind overlapping regions in the base domain and compete, as well. Although this study increased our knowledge of the epitopes of the mAbs in the ZMapp cocktail, the reason for the improved efficacy of ZMapp cocktail still remains as an unknown. Competition assays that investigated the interaction of the ingredients of ZMapp and ZMAb cocktails did not reveal any major differences between 13C6 and 1H3 mAbs, which are the key antibodies that are responsible for the difference in the therapeutic effect of the two cocktails. In conventional competition assays, virus GP is immobilized in a multi-well plate or a sensor surface and reacted with antibody 1 (Ab 1) and antibody 2 (Ab 2) of the competing Ab pair successively. One major disadvantage of this assay format is the inability to identify the antibody interactions with the membrane-associated native GP structure. Moreover, once the Ab 1 is allowed to bind to the immobilized GP to saturation, binding of Ab 2 to the GP is significantly affected by the Ab 1 coverage of the GP. Here, we demonstrate a novel mAb competition assay utilizing SP-IRIS and EBOV-GP carrying whole virus models to evaluate the competition between the six mAbs in the aforementioned three mAb cocktails as well as KZ52 mAb. We reveal the so far unknown competition between the 2G4, 4G7, and 1H3 antibodies. Our competition assay method, which offers a simple, high-throughput, and sensitive platform, can be used to evaluate the therapeutic mAbs developed against other viruses as well, such as SARS-CoV-2 and monkeypox.

## Methods

### Sensor chip preparation

Silicon chips with a thermally grown 100 nm silicon dioxide layer were fabricated by standard silicon processing methods. The chips were cleaned by sonicating in acetone and then rinsing with methanol and Nanopure water. They were dried with nitrogen and treated with oxygen plasma for surface activation for the following polymer coating process. Chips were immersed in 1 × MCP-2 polymer solution (Lucidant Polymers) for 30 min, rinsed with Nanopure water for 5 min and dried with nitrogen. MCP-2 is a 3-D copolymer with NHS groups for covalent biomolecule immobilization. Its chemical composition and advantages over 2-D surface functionalization techniques are described in detail in previously published work^33,34^. Once polymer-coated, SP-IRIS chips were baked at 80 °C for 15 min. Antibodies were spotted on polymer-coated chips using Scienion S3 Flexarrayer piezoelectric arrayer. Antibodies were spotted in PBS with 50 mM Trehalose. During spotting, a humidity level of 57% was maintained in the spotter chamber. The spotted chips were kept in the chamber at 67% humidity overnight. Following the overnight immobilization, the chips were treated with 50 mM Ethanolamine in 1 × Tris—buffered saline (150 mM NaCl and 50 mM Tris—HCl, Fisher Scientific), pH = 8.5, for 30 min to quench the remaining NHS groups in the polymer, then washed with PBST (PBS with 0.1% Tween-20) for 30 min, rinsed with PBS and Nanopure water, and dried with nitrogen.

### Single-particle interferometric reflectance imaging sensor (SP-IRIS): Optical setup and data analysis

**Figure 1:**
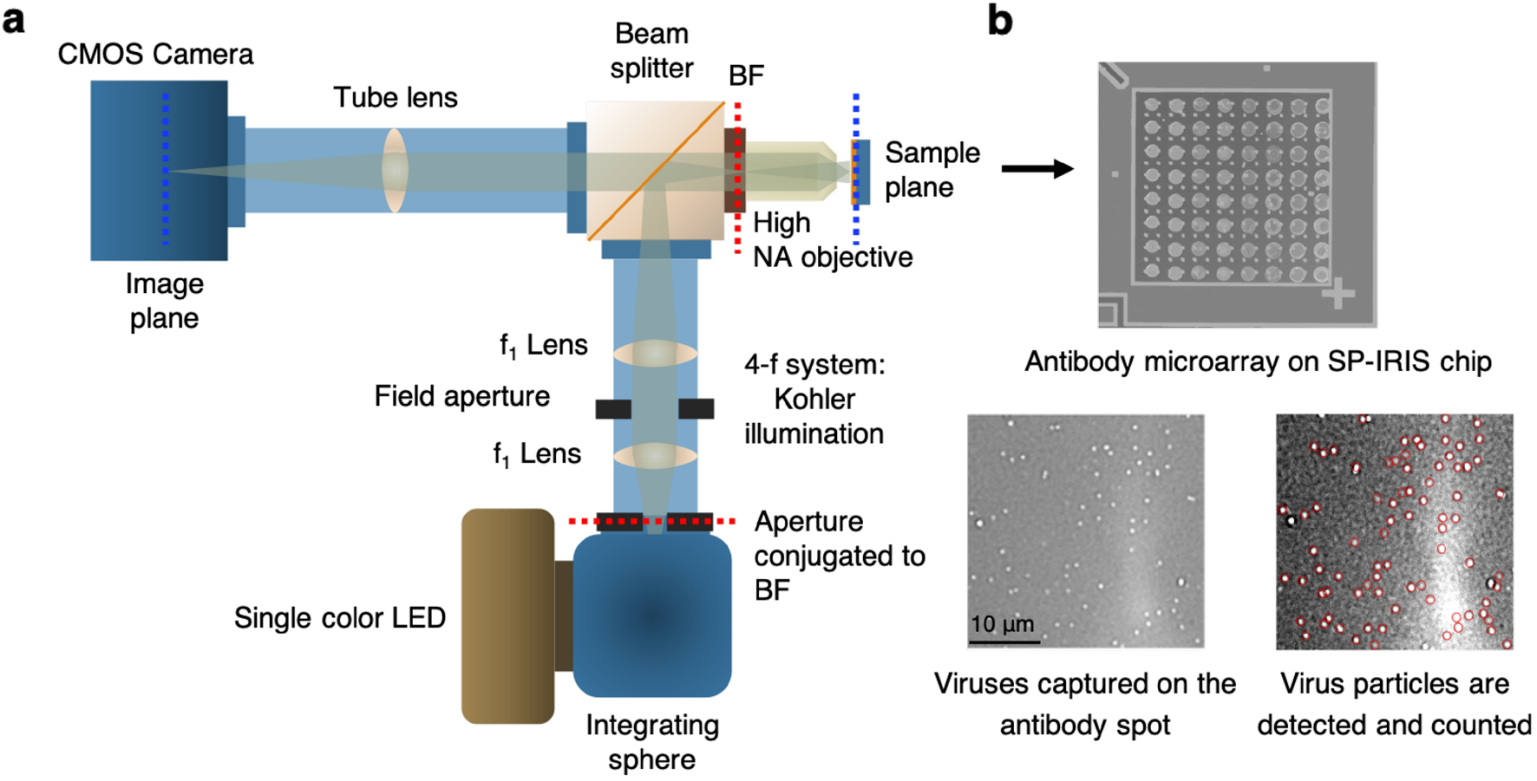
Optical setup of SP-IRIS and virus visualization using SP-IRIS platform. (**a**) Optical setup of SP-IRIS system (BF: Back focal plane) (**b**) Top part shows an SP-IRIS chip with an antibody microarray printed on. The image was taken with low-magnification modality of IRIS system. Each antibody spot is about 150 µm in diameter. Bottom images are zoomed in antibody spots from an SP-IRIS image following an incubation with the specific virus sample. Virus particles appear as white dots and they are detected (red circles) and counted using custom software.

SP-IRIS utilizes a wide-field interferometric imaging technique and a Si/SiO_2_ layered substrate to generate high spatial resolution images of nanoparticles captured on the sensor surface. The substrate illumination is provided by an LED (525 nm) and the image is obtained by a high numerical aperture (NA), high-magnification objective (50 ×, 0.8 NA) and a CCD camera (Fig. 1a). Single particle imaging is achieved by the reflected light that results from the interference between the scattered field from the particles and the reference field reflected from the Si/SiO_2_ interface^7^. The particles captured on the sensor surface appear as diffraction-limited dots in the acquired image and are counted using custom software that identifies particle-associated intensity peaks in the image (Fig. 1b and Supplementary Fig. S1). Any objects in the image that do not correlate with this intensity peak profile such as dust particles or morphological features of the antibody spots are removed using a Gaussian filter. The contrast of the particles is correlated to their size using a forward-model, allowing for size-specific detection to eliminate the background signal^35^. Each antibody spot image is analyzed for the captured particle count and the signal is expressed as particle density (particle count per mm^2^). An image before virus incubation is also taken for each spot to obtain the pre-incubation particle count and this number is subtracted from the post-incubation particle count to calculate the net bound particle count. For screening ebolavirus antibodies, in-liquid SP-IRIS was used where a 30 nm-oxide SP-IRIS chip was mounted on a disposable, multilayer laminate microfluidic cartridge that has an active flow control (Harvard Apparatus, PHD 2000). In-liquid measurements used a 40 ×, 0.9 NA objective and a 3 µl/min of flow rate. Rest of the experiments used dry SP-IRIS measurements with 100 nm-oxide chips where the virus incubation is done in a multi-well plate and the chips are washed and dried prior to chip scanning.

### Screening mAbs against Ebola and Sudan viruses on SP-IRIS

The VSV-based pseudotypes expressing EBOV, SUDV and LASV GPs were generated by inserting the cDNA coding the relevant GP in place of VSV GP in the genome as described previously^36^. Virus sample concentrations were determined by the standard plaque assay method for all of the experiments performed in this study. 13F6, 13C6, 6D8, 2G4, and 4G7 mAbs were provided by Mapp Biopharmaceutical. 1H3 was provided by Public Health Agency of Canada. KZ52 antibody was purchased from IBT Bioservices. 15H10 was provided by Duke University. SUDV-specific mAbs (3F10, 16F6, 5G10, 16H11, 19B3, and 19B4) were provided by U.S. Army Medical Research Institute of Infectious Diseases (USAMRIID). SP-IRIS chips were spotted with three replicate spots for each of these antibodies at a concentration of 3 mg/mL along with a negative control antibody, 8G5, specific for wild-type VSV GP, and the chips were washed as described previously. Next, one SP-IRIS chip was incubated with 10^7^ PFU/mL rVSV-EBOV (prepared in PBS with 1% BSA) and another chip was incubated with 3 × 10^5^ PFU/mL rVSV-SUDV in the microfluidic cartridge for 1 h. Another chip was also incubated with rVSV-LASV at a titer of 10^7^ PFU/mL to determine the specificity of the tested antibodies. At the end of the incubation, spot images were taken with SP-IRIS and analyzed for the average virus densities for each antibody on the three chips.

### Screening mAbs against Lassa virus on SP-IRIS

16 different anti-LASV GP mAbs were provided by Tulane University (Table 2). To test the whole virus capture ability of these antibodies, they were arrayed on SP-IRIS substrates using a spotting concentration of 2–3 mg/ml. Antibodies having lower concentrations than 2 mg/ml were concentrated using a centrifugal filter unit (MWCO = 100 kDa, Amicon). Eight replicate spots were created for each antibody as shown in Fig. 4a. The spotted SP-IRIS chips (2 chips, each with 8 antibodies) were incubated with 10^7^ PFU/ml VSV expressing LASV GP for 1 h (in PBS with 1% BSA) in a multi-well plate. Following incubation, the chips were washed with PBS three times, each 1 min, and then dipped into Nanopure water and dried with nitrogen. Next, the antibody spots were scanned with SP-IRIS and analyzed for bound virus densities on each antibody.

### Specificity and sensitivity of LASV detection using 8.9F mAb

Since 8.9F mAb showed the highest captured virus density in the screening experiment, it was used for the specificity test and limit-of-detection (LOD) experiment to determine the detection sensitivity of rVSV-LASV in complex media. For evaluating the specificity of 8.9F antibody, two SP-IRIS chips were spotted with 8.9F antibody (n = 6 replicate spots) and they were incubated with either 5 × 10^4^ PFU/mL rVSV-LASV or 5 × 10^4^ PFU/mL rVSV-EBOV in PBS with 1% BSA for 1 h. Following the incubation, the chips were washed, dried and imaged using SP-IRIS. Average virus densities were calculated for 8.9F antibody spots on each chip. For the LOD experiment, five SP-IRIS chips were spotted with 8.9F antibody (n = 6 spots) and ten-fold rVSV-LASV dilutions were prepared from 10^6^ PFU/mL stock solution down to 10^3^ PFU/mL using fetal bovine serum (FBS). FBS was purchased from ATCC (#30-2020). Four SP-IRIS chips were incubated with different virus dilutions for 1 h and one blank chip was incubated with FBS only to determine the detection threshold. Chips were washed and analyzed as described previously to obtain the average virus densities on 8.9F antibody spots for each dilution chip.

### Antibody competition assay for anti-EBOV mAbs used in three therapeutic cocktails: MB-003, ZMAb, and ZMapp

Eight SP-IRIS chips were spotted with six anti-EBOV GP mAbs that constitutes the composition of three therapeutic mAb cocktails against Ebola virus infection (13F6, 13C6, 6D8, 1H3, 4G7, 2G4) and an additional mAb, KZ52, that was isolated from a human survivor in the 1995 Zaire EBOV outbreak. 5 × 10^5 ^PFU/ml rVSV-EBOV solution prepared in PBS with 1% BSA was mixed with each of the seven antibodies at a final antibody concentration of 1 μM in separate microtubes and incubated for 20 min to allow the binding of the antibodies to the viruses. Then, each SP-IRIS chip was incubated with one mAb-virus mixture for 1 h and one chip was incubated with rVSV-EBOV alone (reference chip) to calculate the binding levels of the mAbs when there is no competition. At the end of the incubation, the chips were washed and spot images were taken with SP-IRIS. Average virus densities for each mAb (n = 4 replicate spots) were calculated for all of the chips. For the competition chips, the percent binding values were calculated by comparing the signal to the binding level that occurred on the reference chip for each antibody.

## Results and discussion

### Screening mAbs against ebolavirus surface GP on SP-IRIS

To evaluate the performance of the mAbs against EBOV and SUDV GPs on SP-IRIS, we spotted antibodies on three SP-IRIS chips with 3 replicate spots for each antibody and performed virus detection experiments by incubating each chip with a different VSV pseudotype: rVSV-EBOV, rVSV-SUDV, and rVSV-LASV (for evaluating specificity). Our antibody screening test included the ingredients of all three cocktails mentioned in the previous section, 13F6, 13C6, 6D8, 2G4, 4G7, 1H3, as well as additional antibodies: KZ52, 15H10, 3F10, 16F6, 5G10, 16H11, 19B3, and 19B4.

**Figure 2:**
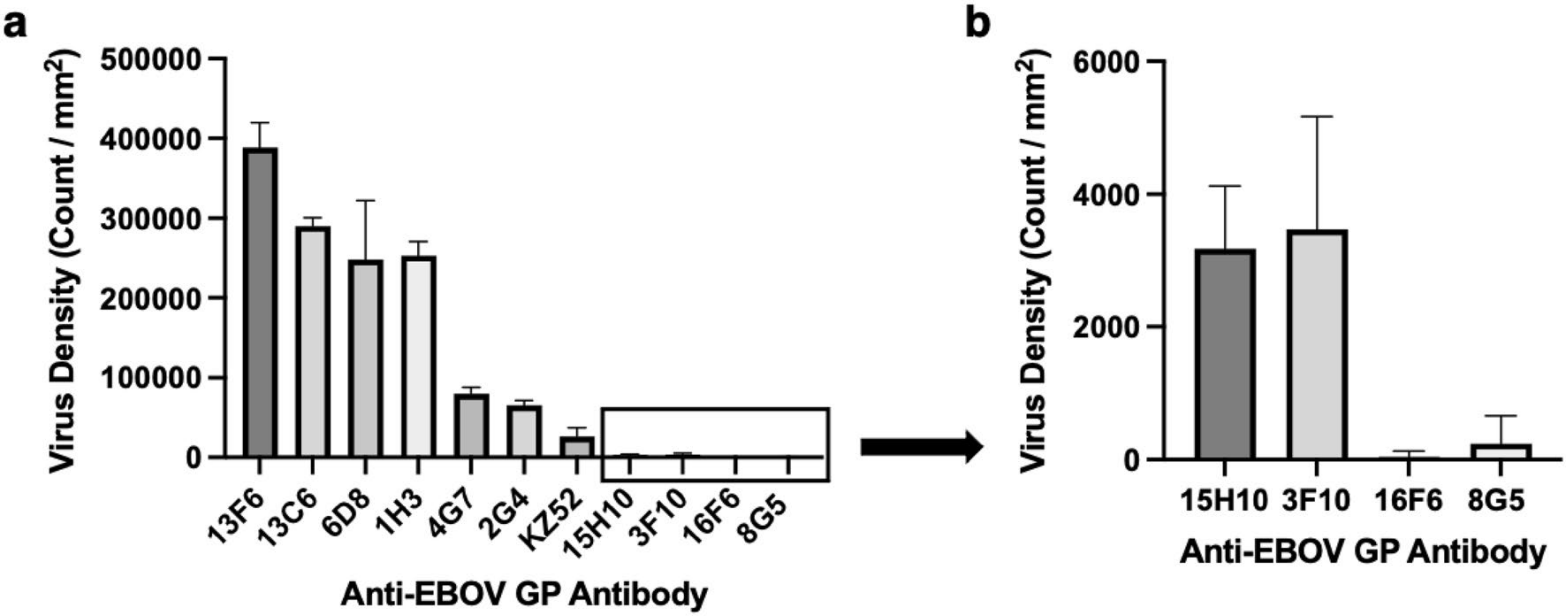
Antibody screening for mAbs against EBOV GP on SP-IRIS using rVSV-EBOV model. (**a**) SP-IRIS signal (virus count/mm^2^) for 10 different mAbs as well as a negative antibody against wild-type VSV (8G5). Average virus densities were calculated from 3 replicate spots for each mAb after the SP-IRIS chip was incubated with 10^7^ PFU/mL rVSV-EBOV for 1 h. (**b**) SP-IRIS signal drawn on a smaller density scale for the last 4 antibodies from (a) to show the signal levels clearly. The detection threshold is calculated from negative 8G5 antibody spots as average virus density plus three times the standard deviation. Any virus density above the detection threshold (2000 particles/mm^2^) is considered as a positive signal whereas antibodies with a lower signal than the threshold are considered to have no binding. Results given in Fig. 2 are representative of at least three independent experiments.

**Figure 3:**
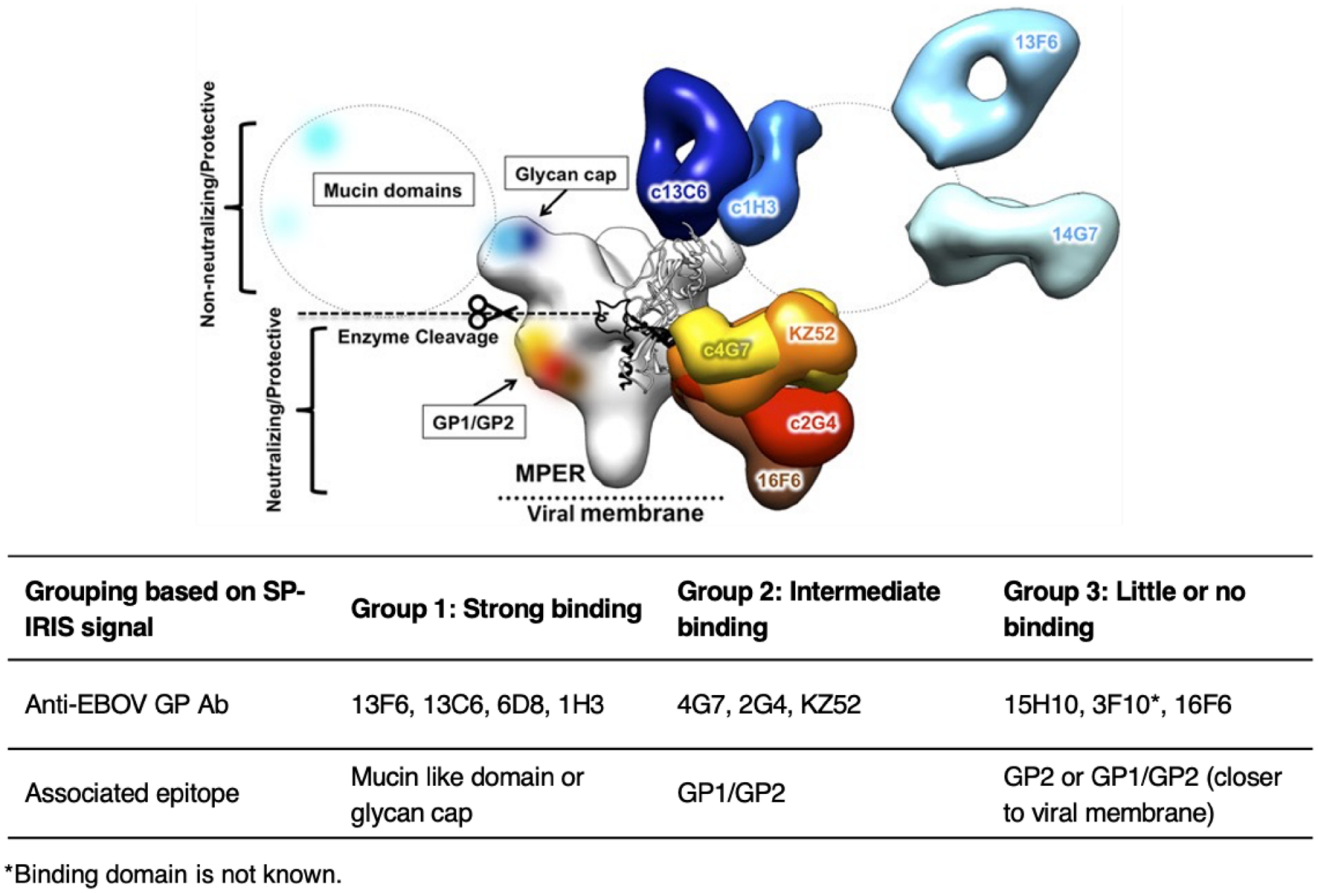
Anti-EBOV GP antibody epitope mapping based on SP-IRIS data. Top image shows the epitopes for protective mAbs on the EBOV GP structure. Reproduced with permission from^32^. The bottom table shows the grouping of mAbs against Ebola virus GP based on their signal levels on SP-IRIS. Group 1 antibodies show the highest level of binding on SP-IRIS with binding sites either on the mucin-like domain or glycan cap, the most accessible areas on the GP for binding. Group 2 antibodies have intermediate binding levels on SP-IRIS and they bind to the GP1/GP2 interface of the GP in the base domain. Group 3 antibodies show little or no binding on SP-IRIS. These antibodies bind either to the base domain with a steeper angle than the Group 2 antibodies or MPER region which is the region of the GP that is the closest to the viral membrane.

According to the binding data obtained from SP-IRIS for the rVSV-EBOV chip (Fig. 2a), the highest amount of virus binding was observed for 13F6, 13C6, 6D8 and 1H3 antibodies, 13F6 antibody giving the highest signal. 13F6 and 6D8 antibodies bind to the mucin-like domain (MLD), and 13C6 and 1H3 antibodies bind to the glycan cap (Fig. 3). Both of these regions are located in the outer part of the GP; therefore, our results can be explained by the easy accessibility of these domains. 2G4, 4G7 and KZ52 antibodies, all of which bind to the base domain of the GP, showed lower signals than the MLD and glycan cap binding mAbs. This may be due to the fact that epitopes of these mAbs are harder to access compared to more exposed MLD and glycan domains. Furthermore, these epitopes might be prevented from mAb binding due to the glycan coating over the GP. Another MLD-specific antibody shown in Fig. 3, 14G7, was not included in our antibody screening experiment since we did not have access to this antibody, however, based on our SP-IRIS data from other MLD-binding antibodies, we expect 14G7 to show high binding levels on SP-IRIS similar to 13F6 and 6D8 antibodies.

KZ52 is a neutralizing mAb that was isolated from a patient who recovered from EBOV infection in the 1995 Zaire EBOV outbreak. This antibody was shown to bind a non-glycosylated region at the base domain of the GP. KZ52 blocks the cleavage of membrane-associated GP by host cell cathepsins, which is a critical step for virus entry into the cell^37^. The cathepsin cleavage results in formation of a fusion-active GP that is capable of mediating membrane fusion. Despite showing similar GP binding affinity to 13C6 in ELISA (Enzyme-linked immunosorbent assay) and SPR measurements performed with purified antigen in a previous work^38^ the capture efficiency of KZ52 was much lower than that of 13C6 on SP-IRIS. The low binding level seen on SP-IRIS with this antibody is most likely due to the decreased accessibility of the base domain of membrane-associated GP combined with the limited flexibility of the surface attached antibodies. KZ52 antibody binds at a parallel angle to the base domain (Fig. 3) and this might pose a sterically hindered orientation for an antibody attached to the surface via multiple bonds.

Figure 2b shows the captured virus densities for the mAbs that showed either very little or no signal on SP-IRIS, as well as the negative antibody, 8G5, specific for wild-type VSV, on a smaller virus density scale to show the signal levels for these antibodies clearly. 15H10 and 3F10 produced very little signal on SP-IRIS whereas there was no significant detection with 16F6 antibody. The detection threshold which is calculated as the mean particle density captured on the 8G5 spots plus three standard deviations, is about 2,000 particles/mm^2^. 15H10 mAb is a non-neutralizing antibody that binds to a site in the canonical heptad repeat 2 (HR2) region near the membrane-proximal external region (MPER) of the glycoprotein (Fig. 3)^39,40^. This antibody has a binding region that is closest to the viral membrane among all the antibodies tested, explaining the low binding level observed on SP-IRIS.

According to data provided by USAMRIID (Supplementary Table S1), 3F10 and 16F6 mAbs showed a positive signal in antigen ELISA for EBOV GP. The fact that these mAbs worked well in an antigen ELISA but did not produce a strong signal for EBOV GP pseudotyped VSV on SP-IRIS platform can be explained by the differences in the accessibility of the specific epitope in the monomeric GP and the trimeric membrane-associated GP. Perhaps the epitopes for these mAbs are hindered from binding due to the organization of the monomers in the trimeric structure. Moreover, 16F6 is a base domain binding antibody, and, as seen in Fig. 3, the angle it binds to the GP is steeper than the rest of the antibodies, making it closer to the viral membrane and putting a constraint on the binding to the GP when the antibody is immobilized on the sensor surface. The binding domain for 3F10 is unknown; however, we predict that it binds either to the base domain or MPER region of GP based on the level of signal obtained on SP-IRIS. Neither 3F10 nor 16F6 mAbs showed binding to the whole virus in flow cytometry (for Zaire species), which is also consistent with the very little signal or lack of signal on SP-IRIS (Supplementary Table S1). Sudan specific antibodies (5G10, 16H11, 19B3, and 19B4) did not show any binding for the chip incubated with rVSV-EBOV (not shown in the graph), as expected, with particle densities well below the threshold.

The SP-IRIS chip that was incubated with rVSV-SUDV showed binding for 3F10, 5G10, and 16F6 antibodies, given in order of decreasing signal. (Supplementary Fig. S2). Other SUDV-specific antibodies, 16H11, 19B3 and 19B4, did not bind to membrane-associated GP of SUDV, although they showed binding in ELISA (Supplementary Table S1). We reason that either the epitopes of these antibodies are not exposed in the GP associated with the viral membrane or the binding angles of the antibodies pose a constraint on the surface-immobilized antibodies. According to our results, 3F10 was the only mAb that recognized both EBOV and SUDV GP in a whole virus model. All three antibodies that were able to bind to rVSV-SUDV on SP-IRIS, 3F10, 5G10, and 16F6, can be used for SUDV detection in whole virus immunoassays, whereas 5G10 would be the most ideal capture antibody to differentiate between Zaire and Sudan species of ebolavirus. Our results demonstrate that SP-IRIS provides a sensitive and fast platform for screening mAbs for different viruses and also different species of a given virus to identify species-specific antibodies as well cross-reacting mAbs. We were able to test 16 different mAbs on a single chip, revealing their binding specificity and relative capture efficiencies. The third chip that was incubated with rVSV-LASV did not show any signal for any of the EBOV and SUDV-specific antibodies whereas there was significant binding for LASV GP specific 8.9F antibody. (Supplementary Fig. S3).

### A model for EBOV GP epitope mapping based on SP-IRIS data

Different experimental techniques have been used to determine the interactions between antibody and antigens. These include X-ray crystallography, nuclear magnetic resonance (NMR), cryo-electron microscopy (cryo-EM), hydrogen–deuterium exchange mass spectrometry (HDX-MS), and ELISA-based synthetic peptide or mutant antigen detection^41,42^. Although X-ray crystallography and NMR are powerful structural biology techniques that can reveal antibody-antigen interactions at the amino acid level, these techniques are not commonly used due to complicated sample preparation, high level of expertise required, and molecular size restrictions^43^. Cryo-EM can provide high-resolution images of large macromolecular assemblies; however, it cannot reach a throughput level required for screening hundreds of different antibodies in a relatively short period of time. In a recent review by Renaud et al. it is mentioned that it would take about half a year of data collection and at least a year of computational analysis to analyze 500 crystals^44^, hampering the utility of this technique for fast characterization of therapeutic antibody candidates.

HDX-MS has recently become a popular tool for characterizing proteins and identifying protein–protein interactions. It is a versatile technique with its ability to work in near-native conditions hence providing insights into protein structure and function^45^. One major limitation of this method is its low throughput especially for analyzing proteins associated with a membrane structure, which is essential for obtaining accurate information about the viral membrane-antibody interface^46^. Therefore, this technique requires further advancements in instrumentation for high-throughput characterization of antibodies against membrane-bound viral antigens. In addition, sample preparation and interpretation of results still remain as challenging tasks in HDX-MS experiments^47^. Besides, even if the epitope information is obtained, it would still be necessary to evaluate the binding properties of antibodies to obtain relative affinities which might provide an insight into their usefulness for mAb-based therapy. For example, for KZ52 antibody, which was identified as a neutralizing base-domain specific antibody, the low binding affinity, which was also observed on SP-IRIS as shown in the previous section, might have played a role in its failure for protection in NHP models.

Other techniques for epitope mapping include use of functional laboratory assays such as ELISA for evaluating the binding of antibodies to either synthetic peptides or GPs that have been modified by site-directed mutagenesis (SDM)^48^. In the case of synthetic peptide detection, antibody-binding amino acid sequences of the virus glycoprotein are identified based on the presence of the signal from a given library of peptide sequences. In SDM-based epitope mapping, mutant GPs that have deleted regions are utilized to understand how the deletion affects binding. Lack of binding means that the deleted region was involved in the epitope. Although ELISA can provide high-throughput analysis, it has limited sensitivity since it can provide a readable signal when only a certain level of binding is achieved. Our SP-IRIS platform, on the other hand, provides single-particle level sensitivity, allowing detection of low affinity antibody-virus GP interactions. In addition, due to its ability to visualize intact virus particles, the binding events captured in SP-IRIS reflect the native-like conditions more accurately than ELISA where a membrane-free form of GP is used. Moreover, SP-IRIS allows use of much less antibody amounts compared to multi-well-format assays due to the microarray nature of its substrates and its compatibility with microfluidics.

Here, we utilize SP-IRIS to provide epitope mapping information based on a predictive model where degree of binding is associated with the binding location on the GP. However, this work can easily be extended to direct identification of epitopes on the GP by using modified GPs created with SDM, showing the potential of SP-IRIS to become a routine tool for high-throughput screening and characterization of mAbs. SP-IRIS can also be used in combination with other techniques such as HDX-MS for cases where more detailed information is needed regarding a specific antibody-antigen interaction.

Based on our results presented in Table 1, we can divide the Ebola virus antibodies (Zaire-specific) that we tested on SP-IRIS platform into three distinct groups (Fig. 3). Group 1 antibodies form the strong binding group and members of this group bind to either the MLD (13F6 and 6D8) or the glycan cap of the GP (13C6 and 1H3). Group 2 antibodies (4G7, 2G4, and KZ52), that show intermediate level of signal on SP-IRIS, bind to the G1/G2 interface (base domain) of GP. The final group, Group 3, is composed of mAbs that show very little or no binding on SP-IRIS. Among these, 15H10 binds to an antigenic site on GP2, HR2/MPER region, which is very close to the viral membrane. 16F6 binds to GP1/GP2 area like Group 2 antibodies, however with a steeper angle and closer proximity towards the viral membrane. 3F10 mAb epitope is not known, however, based on low binding level observed on SP-IRIS, we can hypothesize that it binds to a region close to viral membrane, either to GP1/GP2 or MPER region. Overall, Group 3 antibodies have less accessible binding regions such as MPER and/or steeper binding angles that would be hard to achieve with surface immobilized antibodies due to steric hindrance and limited flexibility.

**Table 1:** mAbs against EBOV GP, their relative signal on SP-IRIS, binding domains on the GP, and neutralization activities.

Anti-EBOV GP Ab	Signal on SP-IRIS	Binding domain	Neutralization activity
13F6	+ + + +	Mucin-like domain	Non-neutralizing
13C6	+ + +	Glycan cap	Non-neutralizing
6D8	+ + +	Mucin-like domain	Non-neutralizing
1H3	+ + +	Glycan cap	Non-neutralizing
4G7	+ +	GP1/GP2 (base)	Neutralizing
2G4	+ +	GP1/GP2 (base)	Neutralizing
KZ52	+ +	GP1/GP2 (base)	Neutralizing
15H10	+	GP2 (HR2/MPER)	Non-neutralizing
3F10	+	Unknown	Non-neutralizing*
16F6	−	GP1/GP2 (base)	Neutralizing*

The degree of binding is denoted by + /- symbols: +  +  +  + very high, +  +  + high, +  + medium, + little, − no binding.

*Neutralization tests for 3F10 and 16F6 were performed by USAMRIID.

Binding domain information also makes it possible to suggest a mechanism of action for a given antibody. For example, if an EBOV antibody is binding to MLD or the glycan cap, this may provide an evidence for the fact that it is a non-neutralizing antibody^32^. Since the mucin-like and glycan domains are cleaved from the GP before host cell membrane fusion, these regions are not associated with neutralization process. Shedlock et al. showed that the cleavage of glycan cap occurred normally in the presence of mucin-like domain binding antibody, 13F6, showing that this antibody does not interfere with the cleavage process^37^. Their work also demonstrated that 13C6 antibody delays the cleavage process, which might contribute to its protective activity. The fact that these antibodies provide protection in vivo can be result of a different mechanism such as antibody dependent cytotoxicity (ADCC). In ADCC, viral particles with GP bound antibodies are recognized by the Fc receptors on the killer cells of the immune system and destroyed. On the other hand, if an antibody binds to the base domain, it is most likely that it has neutralization activity. Neutralization activity can be realized through several different mechanism including prevention of membrane fusion, blocking the attachment to the host cell and aggregation of the viruses. Therefore, based on the model presented in the table in Fig. 3, it is possible to predict the binding domains of the newly identified antibodies and gain insight into neutralization activities and action mechanisms using whole virus models and SP-IRIS platform.

### Screening mAbs against Lassa virus on SP-IRIS and selecting the ideal mAbs for specific and sensitive detection of LASV

**Figure 4:**
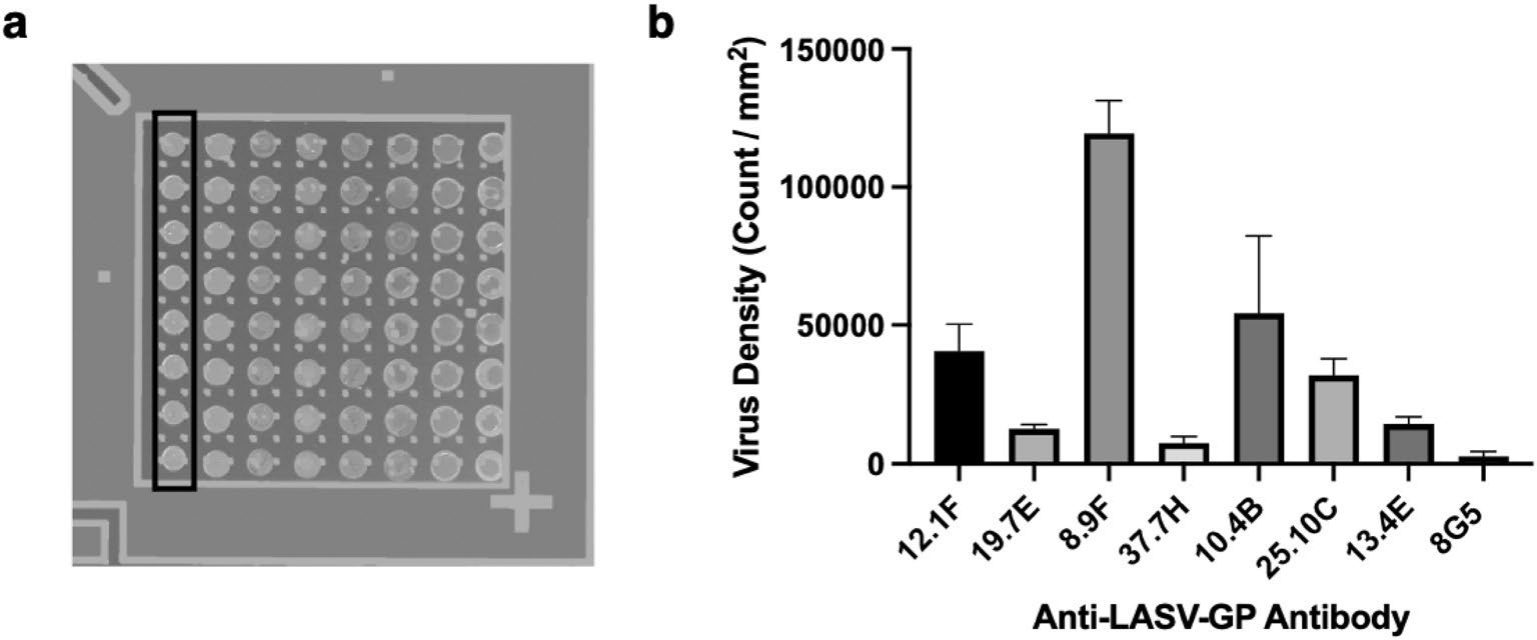
Screening of anti-LASV GP antibodies on SP-IRIS. (**a**) Image of the SP-IRIS chip spotted with eight of the Lassa antibodies. Each column, shown by the black rectangle, represents one antibody type. The rest of the antibodies were spotted on a different chip. Spots have a diameter of approximately 150 μm. (**b**) Average virus densities captured on each antibody for 8 replicate spots following an incubation with 10^7^ PFU/mL rVSV-LASV for 1 h. Wild-type VSV-specific 8G5 antibody was also spotted on the chip to obtain the detection threshold, which is calculated as 7700 particles/mm^2^. Only the antibodies that had a signal over the detection threshold and negative antibody are shown in the bar graph for the sake of simplicity. Results given in Fig. 4 are representative of at least two independent experiments.

To evaluate the whole virus capture efficiency of 16 mAbs against Lassa virus GP, we spotted the following antibodies on two SP-IRIS chips: 8.9F, 10.4B, 12.1F, 25.10C, 13.4E, 19.7E, 37.7H, 2.4F, 37.2D, 36.1F, 19.5A, 4.1F, 18.5C, 9.8A, 37.2G, and 24.6C, with 8 replicate spots for each antibody as shown in Fig. 4a. We incubated both chips with 10^7^ PFU/mL rVSV-LASV for 1 h. The average virus densities for each LASV antibody obtained from SP-IRIS images are shown in Fig. 4b. Only seven antibodies that showed significant binding on SP-IRIS are demonstrated in the bar graph. The antibodies that showed signal on SP-IRIS are 8.9F, 10.4B, 12.1F, 25.10C, 13.4E, 19.7E, and 37.7H. The antibodies that did not show any signal on SP-IRIS are 2.4F, 37.2D, 36.1F, 19.5A, 4.1F, 18.5C, 9.8A, 37.2G, and 24.6C. Since 8.9F showed the highest amount of binding among these, we selected this antibody for the specificity test and LOD experiment for the detection of rVSV-LASV.

**Figure 5:**
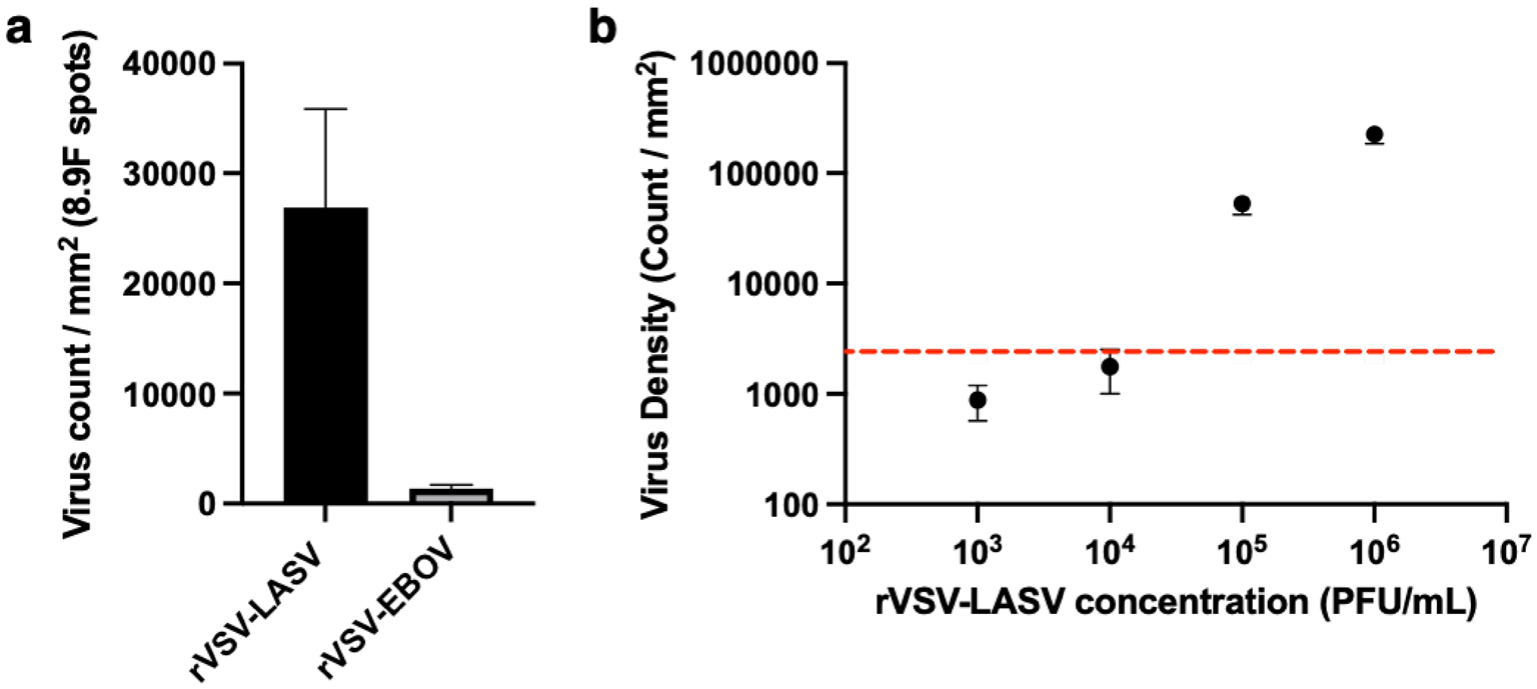
Specificity and sensitivity of 8.9F antibody for rVSV-LASV detection. (**a**) 8.9F antibody specificity test. Average virus densities on 8.9F spots (n = 6 spots) obtained from SP-IRIS images of two different chips incubated with either rVSV-LASV or rVSV-EBOV, both at a concentration of 5 × 10^4^ PFU/mL. 8.9F antibody binds to rVSV-LASV whereas it does not show any significant binding for rVSV-EBOV chip. (**b**) Dilution series experiment for rVSV-LASV detection in FBS using 8.9F as the capture antibody (n = 6 spots). The lowest concentration detected is 10^5^ PFU/mL, however, actual LOD is closer to 10^4^ PFU/mL. Red dashed line represents the detection threshold obtained from 8.9F spots on a chip incubated with FBS alone and calculated as mean virus density plus three standard deviations. LOD experiment was performed at least three times in independent experiments.

To evaluate the specificity of the 8.9F mAb, we incubated two different SP-IRIS chips with different VSV psedotypes: rVSV-LASV and rVSV-EBOV, both at a concentration of 5 × 10^4^ PFU/mL. According to our specificity test results, 8.9F antibody binds to rVSV-LASV whereas it does not show a significant signal for rVSV-EBOV, demonstrating its ability to recognize LASV GP specifically (Fig. 5a). To evaluate the sensitivity of the rVSV-LASV detection in complex media using 8.9F antibody, we performed a dilution series experiment, where we incubated SP-IRIS chips with tenfold dilutions of rVSV-LASV from a 10^6^ PFU/mL stock, prepared in FBS, at the following concentrations: 10^3^, 10^4^, 10^5^ and 10^6^ PFU/mL. Figure 5b shows that the lowest concentration that was detected by SP-IRIS is 10^5^ PFU/mL and the LOD is between 10^4^ and 10^5^ PFU/mL. Given that the specificity experiment that used a 5 × 10^4^ PFU/mL rVSV-LASV showed significant detection (Fig. 5a), we can conclude that the actual LOD is between 10^4^ and 5 × 10^4^ PFU/mL. Red dashed line in Fig. 5b shows the detection threshold obtained from 8.9F spots on a blank chip incubated with FBS alone. It is calculated as the mean of six spots plus three times the standard deviation.

### LASV GP epitope mapping based on SP-IRIS data

**Figure 6:**
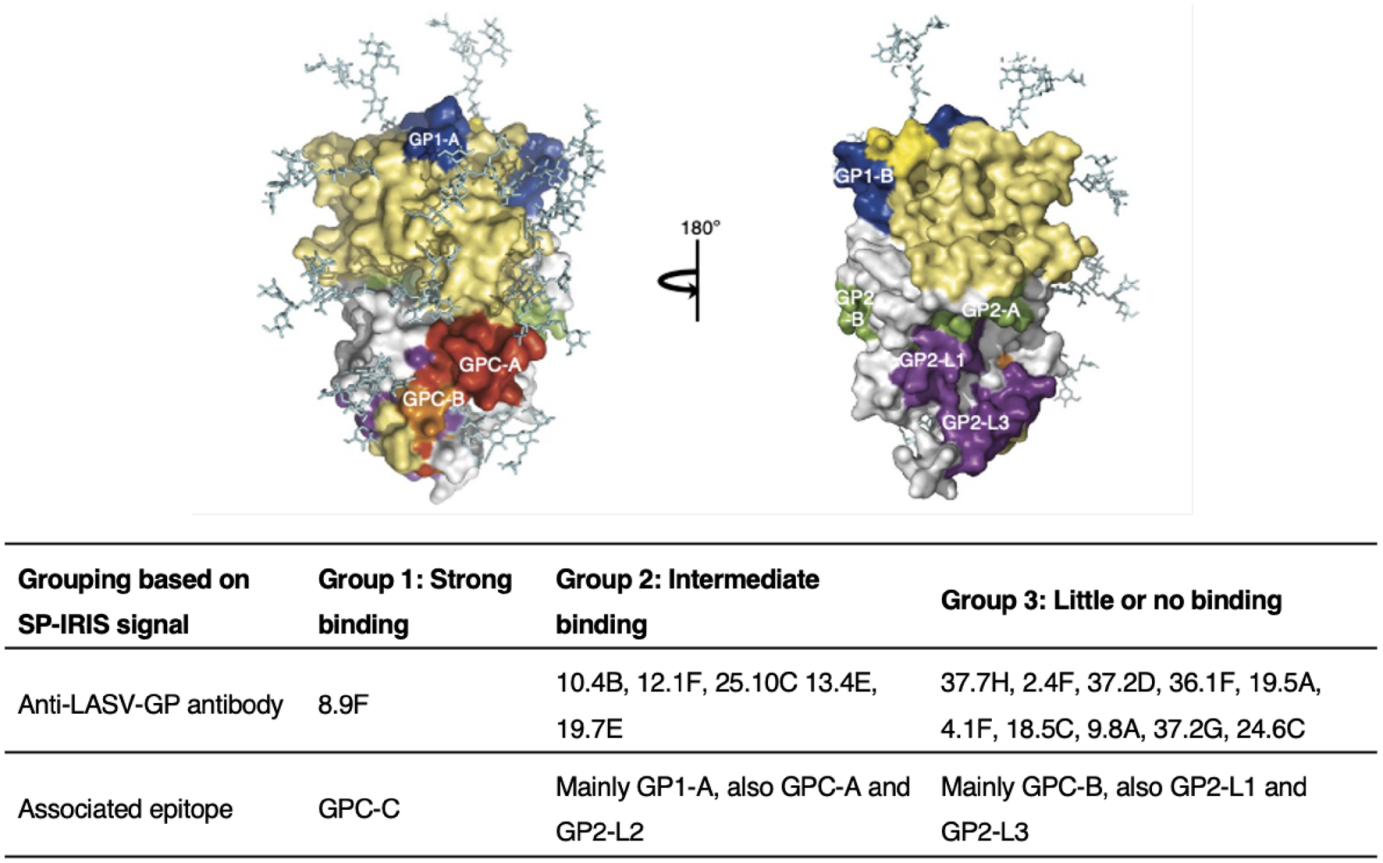
LASV antibody epitope mapping based on SP-IRIS data. Top image shows the surface view of LASV GP monomer structure. GP1 is colored yellow and GP2 is colored white. Epitope color code: GP1-A and GP1-B, blue; GP2-A and GP2-B, green; GP2-L1-3, purple; GPC-A, red; and GPC-B, orange. The location of the conformational epitope of GPC-C, where 8.9F binds, is unknown. Reproduced with permission from ^49^. Bottom table groups the LASV antibodies based on their binding levels on SP-IRIS. The only Group 1 antibody 8.9F binds to a unique region in GPC-C and it is the only antibody that showed a strong binding on SP-IRIS. Group 2 antibodies show intermediate binding on SP-IRIS and mainly binds to the GP1 region of the glycoprotein. Group 3 antibodies, which show little or no binding on SP-IRIS, mostly binds to GPC-B and also GP2-L1 and GP2-L3 linear regions.

In an effort to see if there is a similar correlation between the level of SP-IRIS signal and the binding epitopes on the LASV GP, as we observed with EBOV antibodies, we gathered binding domain information of LASV antibodies from a previously published study^49^. We summarized the relative SP-IRIS signal levels and the binding domains in Table 2 as well as other information about the LASV antibodies including antigen ELISA data and neutralization activities, obtained from the same study^49^. Figure 6 shows a model of LASV GP monomer structure. LASV GP is composed of a glycoprotein complex (GPC) that has two subunits: GP1 and GP2. The 8.9F mAb that showed the highest capture efficiency on SP-IRIS, binds to GPC-C domain that involves both GP units. Among the 16 mAbs tested, 8.9F is the only antibody that binds to GPC-C domain. The fact that 8.9F does not recognize solubilized GP in an ELISA assay (Table 2) suggests that it is reactive against a conformational epitope formed in the membrane-associated GP. All of the other tested LASV antibodies showed signal on ELISA.

**Table 2:** The mAbs against LASV GP tested on SP-IRIS, observed SP-IRIS signal levels, signal on ELISA, binding domains on GP, and neutralization activities.

Anti-LASV Ab	SP-IRIS	ELISA*	Binding domain*	Neutralization activity*
8.9F	+ + +	Negative	GPC-C	Neutralizing
10.4B	+ +	Positive	GP1-A	Weak neutralizing
12.1F	+ +	Positive	GP1-A	Neutralizing
25.10C	+ +	Positive	GPC-A	Neutralizing
13.4E	+ +	Positive	GP2-L2	Non-neutralizing
19.7E	+ +	Positive	GP1-A	Neutralizing
37.7H	+	Positive	GPC-B	Neutralizing
2.4F	−	Positive	GP1-B	Non-neutralizing
37.2D	−	Positive	GPC-B	Neutralizing
36.1F	−	Positive	GPC-A	Neutralizing
4.1 F	−	Positive	GP2-L1	Non-neutralizing
18.5C	−	Positive	GPC-B	Weak neutralizing
9.8A	−	Positive	GPC-B	Weak neutralizing
37.2G	−	Positive	GPC-B	Weak neutralizing
24.6C	−	Positive	GP2-L3	Non-neutralizing

The degree of binding on SP-IRIS is denoted by the number of + symbols: +  +  + high, +  + medium, + little, − no binding.

*Antibody information given in the last three columns was obtained from^49^. 19.5A is not included in the table since there is no information on the binding domain or neutralization activity.

Figure 6 (bottom table) shows the grouping of the LASV antibodies tested in this study according to the signal levels obtained from SP-IRIS and summarizes the correlation between the binding levels and associated epitopes. Group 1 represents the strong binding antibodies and has only one antibody, 8.9F, with a unique binding region in GPC-C. Group 2 is composed of antibodies that show intermediate signal level on SP-IRIS. The majority of the Group 2 antibodies are the ones that bind to GP1-A domain. This is expected since GP1 subunit includes the receptor binding site and also has less glycosylation, making it more accessible for antibody binding. Group 3 is formed by the antibodies that showed either little or no signal on SP-IRIS. Majority of the Group 3 antibodies bind to GPC-B (shown as orange in Fig. 6), which is a region of the GP that is close to the viral membrane. Some antibodies in Group 3 also bind to GP2 linear region (GP2-L1 and GP2-L3). These areas most likely involve the areas of GP2 that are not easily accessible by the antibodies compared to GP2-L2 region. Group 3 antibodies have either a weakneutralizing activity or they don’t have neutralizing activity at all with the exception of 37.2D and 36.1F antibodies (Table 2). Although it is not possible to make a definite remark for a correlation between the SP-IRIS signal and neutralizing activity due to these exceptions, given that there is evidence for neutralizing mAbs to have higher affinities than non-neutralizing ones^49^, we can anticipate the antibodies showing higher signal on SP-IRIS to have higher potency for protecting against the viral infection. According to our results, Group 1 and Group 2 antibodies would be the best candidates to choose as potential therapeutic antibodies against LASV infection. Moreover, identifying more mAbs recognizing the receptor binding site on GP1 subunit which is less glycosylated would help improve the sensitivity of LASV detection. These antibodies can be easily determined on SP-IRIS since they would show high level of binding. Overall, our results demonstrate SP-IRIS as a sensitive and high-throughput platform to screen diagnostic and therapeutic antibody candidates to find the antibodies with the best capture efficiency and highest potential to be effective against the LASV infection.

### Antibody competition assay for Ebola virus specific mAbs

As we mentioned earlier, ZMapp antibody cocktail has been shown to provide increased efficacy compared to the two previously evaluated MB-003 and ZMAb cocktails. ZMapp and ZMAb cocktails have 2G4 and 4G7 antibodies in common and they differ in the third component: ZMAb has 1H3, while ZMapp has 13C6. Therefore, one can conclude that 13C6 has some advantage compared to 1H3 in providing efficient protection. Both of these antibodies bind to the glycan cap and compete with each other. 13C6 binds at a perpendicular angel to the viral membrane whereas 1H3 binds at a less steep angle (Fig. 3).

To explain the superiority of 13C6 antibody over 1H3, we made the following hypothesis: 1H3 antibody hinders binding of either one or both of the base-domain-binding constituents of the ZMAb cocktail, or vice versa, i.e., binding of 2G4 and/or 4G7 antibodies inhibits the binding of 1H3, preventing it from realizing its function of triggering host immune response. Murin et al. reported competition assay results for ZMapp and ZMAb antibodies using ForteBio Octet platform, which is based on biolayer interferometry (BLI)^32^. Competition assay data presented in this work does not reveal any significant differences between 1H3 and 13C6 mAbs in terms of competition with the 2G4 and 4G7 antibodies. According to their data, binding of 4G7 decreases the binding of 1H3 and 13C6 to 41 and 42% of their binding without competition, respectively. Similarly, 2G4 decreases the binding of 1H3 and 13C6 to 48 and 44% of their uncompeted binding, respectively. When antibodies are introduced in the reverse order, both 13C6 and 1H3 affect binding of 2G4 and 4G7 similarly; 13C6 decreases binding of 2G4 and 4G7 to 62 and 51% of their uncompeted binding, respectively, and 1H3 antibody decreases their binding to 74% and 70% of their uncompeted binding, respectively. In fact, their data indicates that 13C6 interferes with 2G4 and 4G7 binding more than 1H3 does, which is not in accordance with the superior effect of 13C6 in the ZMapp cocktail.

**Figure 7:**
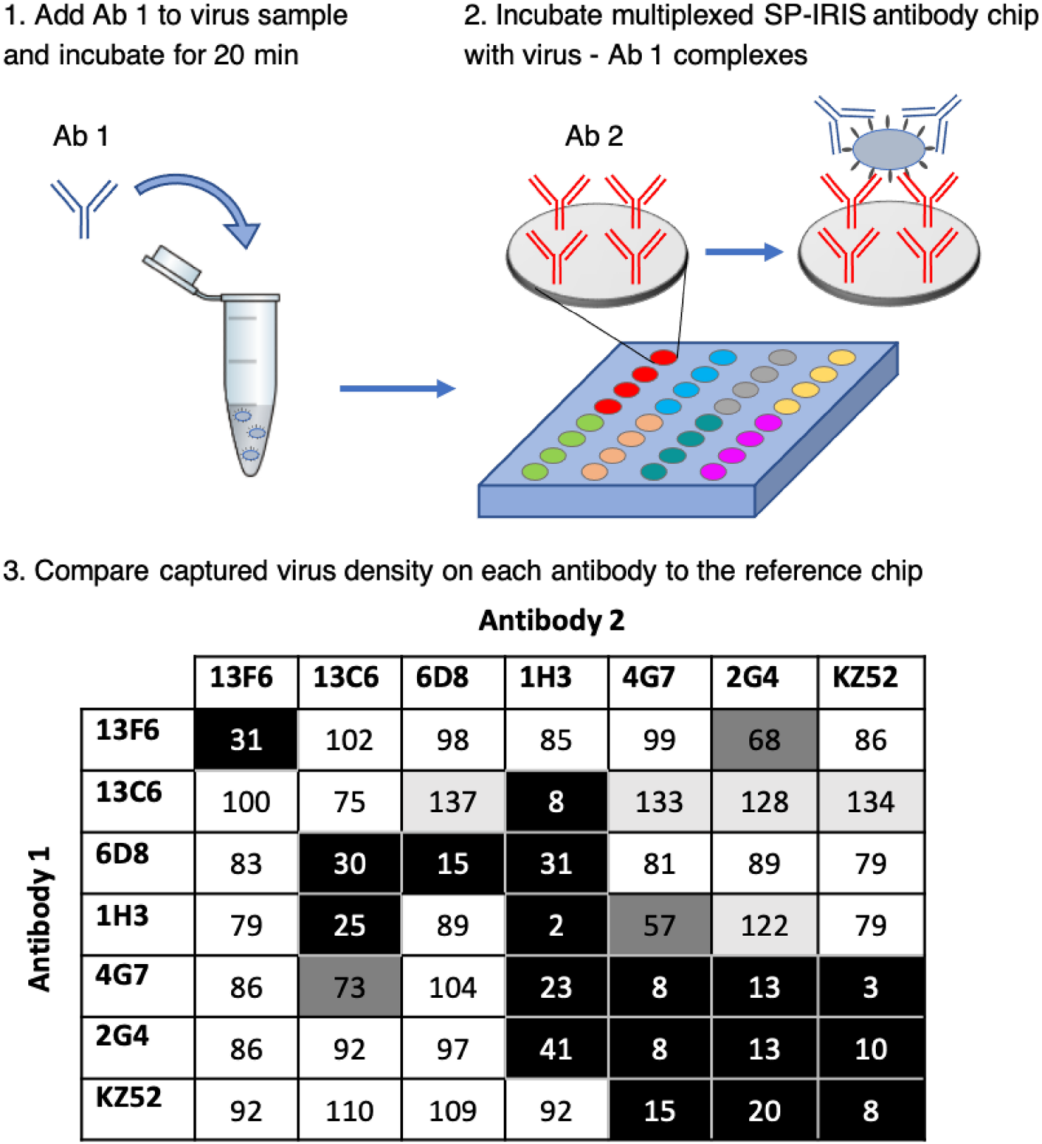
SP-IRIS-based antibody competition assay for anti-EBOV GP antibodies. Top part shows a schematic of the SP-IRIS competition assay. First, antibody 1 (Ab 1) is added to rVSV-EBOV sample in the microcentrifuge tube and incubated for 20 min. Then, this mixture is applied to the SP-IRIS chip spotted with anti-EBOV GP mAbs (Ab 2) and incubated for 1 h. Each mAb is spotted with 4 replicate spots. Following incubation, binding on each mAb is calculated as a percentage of the uncompeted binding from a reference chip that was incubated with the rVSV-EBOV sample alone. Signal on the Ab 2 on the reference chip is taken as 100%. Bottom table summarizes the percent competition binding values. Ab 1 competes with Ab 2 when the percentage is lower than 50% (black boxes). When the percent binding is between 50 and 75%, it is considered intermediate competition (dark gray). Percent binding values between 75 and 110% represent no competition (white boxes), whereas the values over 110% represent an effect that we refer to as binding enhancement (light gray boxes). The results presented in Fig. 7 are representative of two independent competition assays.

We developed a novel competition assay using SP-IRIS platform and pseudoviruses with membrane-associated GP to understand the interaction of the antibodies with the GP in its native state (Fig. 7). We spotted anti-EBOV GP antibodies (13F6, 13C6, 6D8, 1H3, 4G7, 2G4 and KZ52) on eight SP-IRIS chips with four replicate spots per antibody. We first mixed 5 × 10^5 ^PFU/mL EBOV-pseudotyped VSV with each of the seven antibodies (at a final antibody concentration of 1 μM) in separate microtubes and allowed the binding of the antibodies to the rVSV-EBOV particles for 20 min. Then, we incubated seven separate SP-IRIS chips with these mixtures for 1 h. We also incubated one SP-IRIS chip with the virus sample only as the reference chip to calculate the uncompeted binding level of the antibodies. We calculated the average virus densities for each antibody on the surface of each chip and calculated the percent binding for each antibody by comparing the binding on a given competition chip to the uncompeted binding from the reference chip. These percent binding values are given in Fig. 7.

Our results indicate that pre-decoration of virus particles with 4G7 and 2G4 antibodies reduces the binding of 1H3 significantly (to 23 and 41% of its binding alone, respectively). On the other hand, binding of 4G7 or 2G4 to the membrane-associated GP does not interfere with 13C6 antibody binding to the GP significantly, with 73 and 92% competition binding percentages. Based on our results, we predict that binding of 2G4 and 4G7 antibodies causes a conformational change in the region where 1H3 binds, preventing the binding of 1H3, whereas they do not affect binding of 13C6. Therefore, it is possible that, when administered together, 2G4 and 4G7 antibodies will prevent binding of 1H3 to the glycan cap which will hamper the appropriate immune response and decrease the efficacy of treatment. Importantly, when antibody order is reversed, we observed that 13C6 enhances the binding of both 4G7 and 2G4 significantly, with 133% and 128% binding percentages, respectively, whereas 1H3 improves the binding of 2G4 only while reducing the 4G7 binding to 57%. The enhancement effect induced by the 13C6 mAb is likely to result from a conformational change occurring in the 4G7 and 2G4 binding residues. When combined together, our results provide a possible explanation as to why 13C6 antibody in ZMapp cocktail is superior to 1H3 antibody that is in the ZMAb cocktail. 13C6 mAb enhances the binding of other two antibodies in the cocktail, 2G4 and 4G7, and its own binding is not affected significantly by them, rendering this cocktail more efficient than the 1H3, 2G4, and 4G7 mixture. These results suggest that competition between the antibodies in a cocktail has important implications for the efficacy of the treatment, and therefore should be carefully evaluated while designing mAb cocktails. Moreover, our data showing the affinity-enhancing effect of 13C6, highlights the importance of blending different mAbs in a therapeutic antibody mixture. In addition to combining different mechanisms of action, the mixing of different mAbs might potentially increase the binding affinity of certain mAbs in the mixture and hence their efficacy. 13C6 also showed a high binding percentage when it is competing with itself. This cannot be due to incomplete saturation of the viral GP, since 13C6 effectively reduced the binding of 1H3, which has a binding region that overlaps with 13C6. These results point to an exceptional binding capacity of 13C6, not typical of a mAb capable of binding to a single epitope on the GP. This unique binding property of 13C6 might have also contributed to the increased efficacy of ZMapp mixture. Table 3 classifies the Ebola antibodies evaluated in the competition assay into different competition groups based on the percent binding levels on the competition chips. We suggest that mAbs in a given antibody cocktail that is being developed as an antiviral agent should be tested pairwise to see the effect of each mAb on the binding of the others. The mAb pairs that are from Group 3 and/or Group 4 would be ideal candidates for further evaluation in clinical trials. For example, KZ52 mAb, which was not effective when administered alone in NHP models, can be combined with 13C6 mAb, which increased its binding by 34% according to our results. Furthermore, one MLD-binding antibody, such as 13F6, can be added to this mixture to potentially trigger different immune response mechanisms without competing with KZ52 or 13C6. 6D8 mAb, despite being an MLD-binding antibody, would not be suitable for this combination since it decreases the binding of 13C6 significantly.

**Table 3:** Classification of Ebola antibodies in the competition assay into competition groups based on their percent binding levels.

Competition group	Binding percentage	Classification
Group 1 (black)	< 50%	Competing
Group 2 (dark gray)	50–75%	Intermediate competition
Group 3 (white)	75–110%	Non-competitive
Group 4 (light gray)	> 110%	Enhancing

The fact that our competition assay results are different than the ones obtained in the previously published study can be explained by the major differences in the two competition assay designs. First of all, in the mentioned study^32^, BLI, which is an ensemble-based method, is used where surface immobilized soluble GP is reacted with the competing antibody pair successively. Ensemble-based techniques have limited sensitivities since they cannot resolve single binding events. On the other hand, SP-IRIS is a highly sensitive platform with single-particle visualization ability, making the competition assay more sensitive. Second, binding of the first antibody to the surface immobilized GP would decrease the binding of second antibody substantially, since GP has limited accessibility due to surface attachment and Ab 1 coverage. This may also explain why the enhancement affect is not observed in the cited work and why 13C6 binding is decreased substantially by 4G7 and 2G4 binding. Moreover, the soluble version of the GP would have different binding properties compared to the viral membrane-integrated GP used in our assay and, therefore, might not reveal the competition and enhancement events that are more likely to occur in native conditions. Thus, our competition assay design provides significant advancements over the existing techniques, creating a novel application for SP-IRIS and making it an excellent candidate for routine characterization process for antibody-based therapeutic development.

## Conclusions

Capture antibody selection is an important design consideration for solid-phase immunoassays. We utilized SP-IRIS for identifying specific mAbs with high capture efficiencies for the detection of VSV-based models of EBOV, SUDV, and LASV. Combined with the binding affinity data, virus binding level on surface-immobilized antibodies on SP-IRIS might provide insights into the antibody epitopes on the glycoprotein structure. mAbs that bind to glycan cap or MLD of the EBOV GP show a high level of signal on SP-IRIS whereas base domain binding antibodies have substantially less capture efficiency. Therefore, high amount of binding on SP-IRIS for a given anti-EBOV GP antibody can be associated with glycan cap or MLD specificity of that antibody. These antibodies could be potential candidates for being protective antibodies against Ebola virus infection.

We also evaluated the competition between the therapeutic mAbs against EBOV surface GP. Our innovative experimental approach combined a highly sensitive, single particle detection platform, SP-IRIS, with pseudovirions that express the native GP, generating a novel competition assay. Our experimental design, where the virus solution was first mixed and incubated with the antibody 1 and then introduced to the multiplexed antibody chip, allowed us to reveal a competition that was unknown so far. Our approach can test one antibody’s competition against tens of antibodies spotted on the same chip, providing a simple and quick assay. Moreover, it requires only one reference chip, whereas the traditional competition assay requires one well per antibody pair tested and a reference well for each antibody. Our competition assay that revealed the competition between seven antibodies took a total of 4 h including pre- and post-incubation scans, two antibody incubation steps and analysis time. Currently, it is possible to run the binding experiments in real-time and obtain results at the end of the incubation period with no further processing. We believe that the scientific community will greatly benefit from our approach of combining SP-IRIS platform and use of whole virus particles for obtaining sensitive and accurate competition information for mAbs. This application of SP-IRIS will be particularly valuable for revealing the competition between antibodies that are candidates for use in therapeutics, enabling the design of efficient cocktails for use in post-exposure therapy.

## Supplementary Information

Supplementary Information.

## Data Availability

The datasets generated during the current study are available from the corresponding author on reasonable request.

## References

[1] COVID-19 Map— Johns Hopkins Coronavirus Resource Center. https://coronavirus.jhu.edu/map.html. Accessed: 14th July 2022.

[2] Multi-country monkeypox outbreak: situation update. https://www.who.int/emergencies/disease-outbreak-news/item/2022-DON396. Accessed: 14 July 2022.

[3] Sharma A (2021). Optical biosensors for diagnostics of infectious viral disease: A recent update. Diagnostics.

[4] Pal M, Das V, BoruahDeka HPDHP, Chikkaputtaiah C (2022). Multiplexed biosensors for virus detection. Adv. Biosens. Virus Detect. Smart Diagn. Combat SARS-CoV-2.

[5] Daaboul GG (2010). High-throughput detection and sizing of individual low-index nanoparticles and viruses for pathogen identification. Nano Lett..

[6] Daaboul GG (2014). Digital sensing and sizing of vesicular stomatitis virus pseudotypes in complex media: A model for ebola and marburg detection. ACS Nano.

[7] Avci O, Ünlü NL, Özkumur AY, Ünlü MS (2015). Interferometric reflectance imaging sensor (IRIS)—A platform technology for multiplexed diagnostics and digital detection. Sensors (Switzerland).

[8] Monroe MR (2013). Single nanoparticle detection for multiplexed protein diagnostics with attomolar sensitivity in serum and unprocessed whole blood. Anal. Chem..

[9] Marn AM, Chiodi E, Ünlü MS (2021). Bulk-effect-free method for binding kinetic measurements enabling small-molecule affinity characterization. ACS Omega.

[10] Zhou X, Zhang L, Pang W (2016). Performance and noise analysis of optical microresonator-based biochemical sensors using intensity detection. Opt. Express.

[11] Squires TM, Messinger RJ, Manalis SR (2008). Making it stick: Convection, reaction and diffusion in surface-based biosensors. Nat. Biotechnol..

[12] Daaboul GG (2016). Digital detection of exosomes by interferometric imaging. Sci. Rep..

[13] EkizKanik F (2022). Attomolar sensitivity microRNA detection using real-time digital microarrays. Sci. Rep..

[14] Sevenler D, Trueb J, SelimÜnlü M (2019). Beating the reaction limits of biosensor sensitivity with dynamic tracking of single binding events. Proc. Natl. Acad. Sci. U. S. A..

[15] Scherr SM (2016). Real-time capture and visualization of individual viruses in complex media. ACS Nano.

[16] Scherr SM (2017). Disposable cartridge platform for rapid detection of viral hemorrhagic fever viruses. Lab Chip.

[17] Seymour E, Ünlü NL, Carter EP, Connor JH, Ünlü MS (2021). Configurable digital virus counter on robust universal DNA chips. ACS Sensors.

[18] Salazar G, Zhang N, Fu T-M, An Z (2017). Antibody therapies for the prevention and treatment of viral infections. npj Vaccines.

[19] Lai SK, McSweeney MD, Pickles RJ (2021). Learning from past failures: Challenges with monoclonal antibody therapies for COVID-19. J. Control. Release.

[20] Maruyama T (1999). Ebola virus can be effectively neutralized by antibody produced in natural human infection. J. Virol..

[21] Wilson JA (2000). Epitopes involved in antibody-mediated protection from Ebola virus. Science.

[22] Takada A, Ebihara H, Jones S, Feldmann H, Kawaoka Y (2007). Protective efficacy of neutralizing antibodies against Ebola virus infection. Vaccine.

[23] Qiu X (2011). Characterization of Zaire ebolavirus glycoprotein-specific monoclonal antibodies. Clin. Immunol..

[24] Oswald_2007_KZ52_failure_in_NHPs.pdf.

[25] Marzi A (2012). Protective efficacy of neutralizing monoclonal antibodies in a nonhuman primate model of ebola hemorrhagic fever. PLoS ONE.

[26] Qiu X (2012). Ebola GP-specific monoclonal antibodies protect mice and guinea pigs from lethal Ebola virus infection. PLoS Negl. Trop. Dis..

[27] Qiu X (2012). Successful treatment of Ebola virus-infected cynomolgus macaques with monoclonal antibodies. Sci. Transl. Med..

[28] Olinger GG (2012). Delayed treatment of Ebola virus infection with plant-derived monoclonal antibodies provides protection in rhesus macaques. Proc. Natl. Acad. Sci. U. S. A..

[29] Pettitt J (2013). Therapeutic intervention of ebola virus infection in rhesus macaques with the MB-003 monoclonal antibody cocktail. Sci. Transl. Med..

[30] Qiu X (2014). Reversion of advanced Ebola virus disease in nonhuman primates with ZMapp. Nature.

[31] Randomized A (2016). Controlled trial of ZMapp for ebola virus infection. N. Engl. J. Med..

[32] Murin CD (2014). Structures of protective antibodies reveal sites of vulnerability on ebola virus. Proc. Natl. Acad. Sci. U. S. A..

[33] Cretich M, Pirri G, Damin F, Solinas I, Chiari M (2004). A new polymeric coating for protein microarrays. Anal. Biochem..

[34] Pirri G, Damin F, Chiari M, Bontempi E, Depero LE (2004). Characterization of a polymeric adsorbed coating for DNA microarray glass slides. Anal. Chem..

[35] Trueb JT, Avci O, Sevenler D, Connor JH, Ünlü MS (2017). Robust Visualization and Discrimination of Nanoparticles by Interferometric Imaging. IEEE J. Sel. Top. Quantum Electron..

[36] Mire CE (2015). A single-vector, single-injection trivalent filovirus vaccine: Proof of concept study in outbred guinea pigs. J. Infect. Dis..

[37] Shedlock DJ (2010). Antibody-mediated neutralization of Ebola virus can occur by two distinct mechanisms. Virology.

[38] Zhang Q (2016). Potent neutralizing monoclonal antibodies against Ebola virus infection. Nat. Publ. Gr..

[39] Yu JS (2006). Detection of Ebola virus envelope using monoclonal and polyclonal antibodies in ELISA, surface plasmon resonance and a quartz crystal microbalance immunosensor. J. Virol. Methods.

[40] Flyak AI (2018). Broadly neutralizing antibodies from human survivors target a conserved site in the ebola virus glycoprotein hr2-mper region. Nat. Microbiol..

[41] Abbott WM, Damschroder MM, Lowe DC (2014). Current approaches to fine mapping of antigen-antibody interactions. Immunology.

[42] Wigge C, Stefanovic A, Radjainia M (2020). The rapidly evolving role of cryo-EM in drug design. Drug Discov. Today Technol..

[43] Nilvebrant J, Rockberg J, Rockberg J, Nilvebrant J (2018). An introduction to epitope mapping. Epitope Mapping Protocols.

[44] Renaud JP (2018). Cryo-EM in drug discovery: Achievements, limitations and prospects. Nat. Rev. Drug Discov..

[45] Opuni KFM (2018). Mass spectrometric epitope mapping. Mass Spectrom. Rev..

[46] Watson MJ (2021). Simple platform for automating decoupled LC-MS analysis of hydrogen/deuterium exchange samples. J. Am. Soc. Mass Spectrom..

[47] Ozohanics O, Ambrus A (2020). Hydrogen-deuterium exchange mass spectrometry: A novel structural biology approach to structure, dynamics and interactions of proteins and their complexes. Life.

[48] Rojas G, Ossipow V, Fischer N (2014). Fine epitope mapping based on phage display and extensive mutagenesis of the target antigen. Monoclonal Antibodies: Methods and Protocols.

[49] Robinson JE (2016). Most neutralizing human monoclonal antibodies target novel epitopes requiring both Lassa virus glycoprotein subunits. Nat. Commun..

