# Supplementary material for: A high-throughput single-particle imaging platform for antibody characterization and a novel competition assay for therapeutic antibodies
Source: Sci Rep. 2023 Jan 6;13:306. doi: 10.1038/s41598-022-27281-w (PMC9821353; doi:10.1038/s41598-022-27281-w)
Supplement: Supplementary file 1 — Supplementary Information. [file 41598_2022_27281_MOESM1_ESM.pdf]

A high-throughput single-particle imaging platform for antibody characterization and a novel competition assay for therapeutic antibodies

Elif Seymour,[†,‡] *M. Selim Ünlü,*[†, §] *John H. Connor*[*,ǁ]

[†]Department of Biomedical Engineering, Boston University, Boston, Massachusetts 02215, USA

[‡]Lunenfeld-Tanenbaum Research Institute, Sinai Health System, Toronto, ON, M5G 1X5, Canada

[§] Department of Electrical and Computer Engineering, Boston University, Boston, Massachusetts 02215, USA

[ǁ]Department of Microbiology, Boston University School of Medicine, Boston, Massachusetts 02118, USA

[*]John H. Connor: Phone: 617-358-9155, Email: jhconnor@bu.edu

Supplementary Information

Schematic representation of Interferometric Reflectance Imaging Sensor (IRIS):

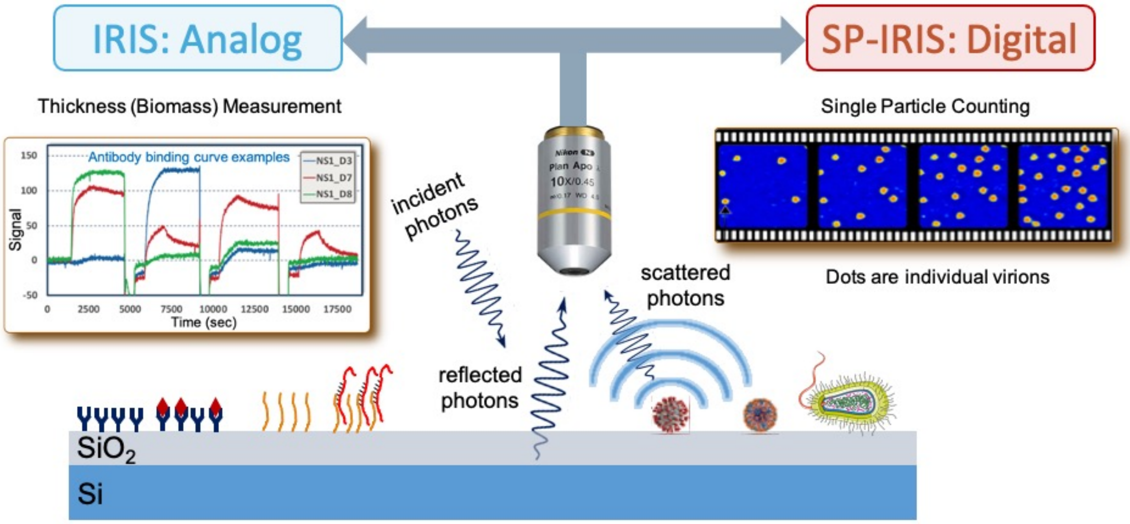

Figure S1: Interferometric reflectance imaging sensor (IRIS) technology is based on interference of light from an optically transparent thin film. IRIS has two modalities: (i) low-magnification (analog detection for ensemble-based biomolecular mass measurements) allowing for multiplexed affinity measurements and (ii) high-magnification allowing for digital detection of individual nanoparticles.

In analog detection (Fig. S1, left), ensemble binding of biomolecules on the sensor surface is detected as a thickness increase by monitoring small optical reflectivity changes. When nano and micro particles, such as viruses and bacteria are captured on the sensor, they are detected as individually discernible events leading to digital counting of bound particles (Fig. S1, right). This modality is called single-particle IRIS (SP-IRIS). In the current study, SP-IRIS setup with either a 50× (dry measurements) or 40× objective (in-liquid measurements) was used although in new generation SP-IRIS system, it is possible to use a 10× objective allowing for a larger field of view and substantially less chip scan time.

Screening anti-EBOV GP and anti-SUDV GP antibodies on SP-IRIS using rVSV-SUDV and rVSV-LASV: Identifying SUDV-specific antibodies and evaluating cross-reactivity

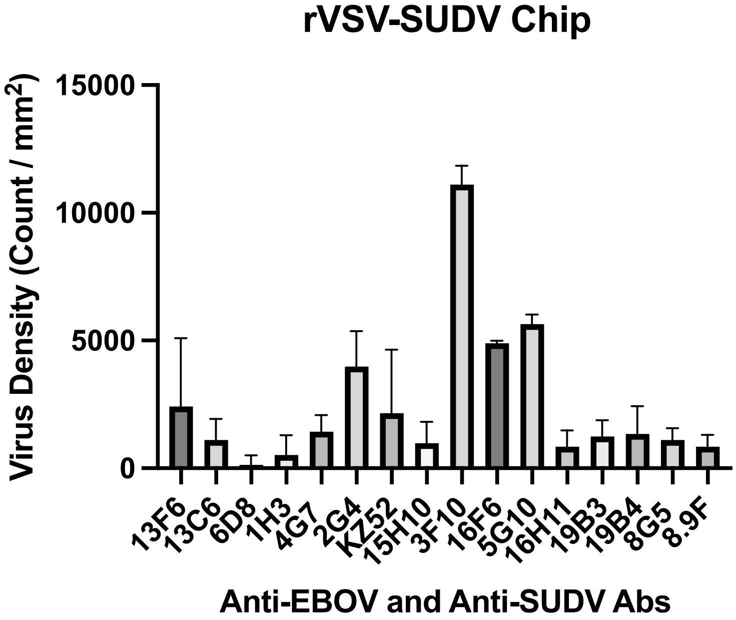

Figure S2: Captured virus densities on EBOV and SUDV specific antibodies for the rVSV-SUDV chip. One SP-IRIS chip spotted with all anti-EBOV and anti-SUDV GP antibodies was incubated with 3 x 10^5 PFU/mL rVSV-SUDV sample for 1 h in the microfluidic cartridge, and then scanned with SP-IRIS. Average virus densities were calculated from 3 replicate spots for each mAb. 8G5 (wild-type VSV-specific) and 8.9F (LASV-specific) antibodies were spotted as negative controls to evaluate the specificity and also to calculate the detection threshold (mean virus density from 8G5 spots plus three times the standard deviation). The threshold for detection for this chip is 2,500 particles/mm^2.

According to Figure S2, among SUDV specific antibodies, 3F10, 16F6, and 5G10 mAbs showed binding to SUDV GP pseudotyped VSV, whereas there was no signal with 16H11, 19B3, and 19B4 antibodies. Also, one of the EBOV specific (Zaire species) mAbs, 2G4, showed minimal signal for rVSV-SUDV, with a virus density of about 4,000 particle/mm^2.

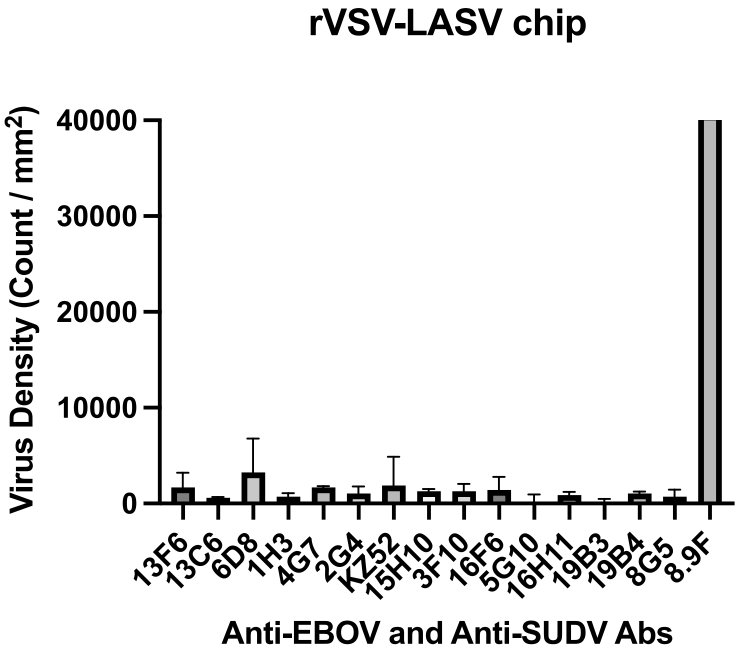

Figure S3: Captured virus densities on EBOV and SUDV specific antibodies for the rVSV-LASV chip. The y scale has been adjusted to show the densities for EBOV and SUDV-specific antibodies more clearly. The virus density on 8.9F spots is 439,112 particles/mm^2. The detection threshold for this chip is 2,900 particles/mm^2.

Figure S3 shows the bound virus densities on anti-EBOV and anti-SUDV GP antibodies for the SP-IRIS chip that was incubated with 10^7 PFU/mL rVSV-LASV sample. The only antibody that showed significant binding is LASV GP-specific mAb, 8.9F. There is a minimal cross-reactivity from 6D8 with 3,253 particles/mm^2, just above the threshold. For this antibody, only 1 spot out of 3 showed a value over the detection threshold. Moreover, the virus titer used in this experiment is very high which makes it more likely to have non-specific binding. Therefore, 6D8 binding can be considered as insignificant.

Table S1: Comparison of ELISA, SP-IRIS, and flow cytometry signals for SUDV-specific antibodies.

Anti-SUDV Abs	Signal on ELISA*	Signal on SP-IRIS	Flow Cytometry*
3F10	Sudan and Zaire	Sudan and Zaire	Negative
16F6	Sudan and Zaire	Sudan	Sudan
5G10	Sudan	Sudan	Negative
16H11	Sudan	-	Negative
19B3	Sudan	-	Negative
19B4	Sudan	-	Negative

*ELISA and flow cytometry data were provided by USAMRIID.
